# Knowledge, Attitude, and Practices towards Malaria among Employees from Enterprises in the Town of Douala, Cameroon

**DOI:** 10.1155/2020/8652084

**Published:** 2020-06-26

**Authors:** Christian Mbohou Nchetnkou, Loick Pradel Kojom Foko, Leopold Gustave Lehman

**Affiliations:** ^1^Department of Animal Organisms, Faculty of Science, The University of Douala, P.O. Box 24157, Douala, Cameroon; ^2^Department of Biological Sciences, Faculty of Medicine and Pharmaceutical Sciences, The University of Douala, P.O. Box 24157, Douala, Cameroon

## Abstract

**Background:**

Malaria remains a public health issue in the world especially in resource-limited countries, where it has a negative impact on their economy. There is a body of recent reports outlining the crucial role of enterprises in control of malaria. The present study aimed at determining the knowledge, attitudes, and practices (KAP) towards malaria among employees from enterprises in the town of Douala, Cameroon.

**Methods:**

A cross-sectional descriptive study took place between February 2015 and June 2017 in 14 enterprises of Douala. A pretested structured questionnaire was used to document sociodemographic parameters and KAP of employees.

**Results:**

A total of 2705 employees, mainly consisted of males (83.1%), were included in the study. The mean age of respondents was 37.33 ± 9.78 years (range 19-87). Over 90% of respondents knew at least one sign/symptom (94.1%) and associated malaria transmission with mosquito bites (91%). Artemether-Lumefantrine (36.2%), under commercial name “Coartem,” was the most cited antimalarial drug. Nearly 98.2% considered malaria as a dangerous disease. Misconceptions on malaria transmission, causative agent, prevention, and treatment were recorded. More than 77% of employees declared to use mosquito bed nets to prevent malaria. About 63% employees declared going to the hospital when they were feel having malaria while 12.9% were seeking care through street drugs. Educational level, socioprofessional category, area of residence, and enterprise were significantly associated with the level of knowledge on malaria transmission, causative agent, and preventive and treatment practices.

**Conclusion:**

This study showed a good level of knowledge, attitudes, and practices of employees even though some misconceptions and bad behaviors are still present especially in people with a low level of education. Hence, there is a need to develop strategies for sensitization especially in this fraction of employees. In addition, enterprises could be an interesting approach in order to control malaria in Cameroon.

## 1. Introduction

Malaria remains a major public health concern across the world. According to the World Health Organization (WHO), an estimated 228 million cases of malaria and 405,000 deaths occurred worldwide in 2018 [1]. Sub-Saharan Africa (SSA) alone accounted for 93% of cases and 94% of deaths. In Cameroon, malaria is the first cause of consultation (26%) and hospitalization (46%); it is responsible for 22% of annual deaths [2].

Malaria has a negative impact in many sectors, including education [3], agriculture [4, 5], and business [6]. Roll Back Malaria (RBM) consortium reported that malaria has negative effect on enterprises in SSA [7]. Malaria negatively impacts enterprises. It causes a decrease in productivity and investments due to both increased employee absenteeism and health care expenditures [7]. Previous studies in Equatorial Guinea, Zambia, Ghana, have revealed that enterprises could play an important role in the reduction of malaria incidence through the integration and implementation of malaria control strategies in their management policies [7, 8]. These strategies include the distribution of long-lasting insecticide-treated nets (LLINs), insecticide residual spraying (IRS) campaigns, and the implementation of rapid malaria diagnosis. The implementation of the above-mentioned control strategies has resulted in significant reduction in malaria burden not only in employees but also in communities in which the enterprises are installed [7].

Malaria control is changing in several endemic countries where community/individuals adhesion to control and prevention measures is placed at the core of control policies, with a growing interest in health education through the understanding of knowledge, attitudes, and practices (KAP) towards malaria and its control [9]. KAP studies are also essential in establishing epidemiological and behavioral baselines in monitoring programs against malaria [9]. In fact, a person who has poor KAP towards a given disease may probably be reluctant to use some of the preventive measures and communicate the information on their positive effects on the control of the disease. Furthermore, this person may get involved in poor practices such as self-medication and improper use of LLINs [3, 10]; they could transmit their misconceptions to relatives. It is therefore critical to monitor the knowledge, attitudes, and practices of the population towards malaria.

A recent study on malaria in some enterprises in Cameroon revealed 30.1% prevalence of the infection among employees [11]. Given the magnitude of the disease and its potential negative impact on businesses [12], enterprises should better commit in malaria prevention and control [13, 14]. Investigating KAP of employees would generate evidence for the development of malaria control and prevention strategies of their employees.

This study was conducted in companies with the aim of generating evidence that would serve as a basis for the development and revision of control strategies, adapted to the enterprises and communities that host them.

## 2. Materials and Methods

### 2.1. Type and Study Site

The study was conducted in Douala (Cameroon), the economic capital city of the country, accounting for 33.5% of enterprises [15]. Fourteen enterprises are from 12 sectors of activity, having their headquarters in Douala. Douala has a warm and humid climate with a mean temperature of ~26°C and heavy rainfall, especially during the rainy season that goes from June to October [16]. This climate is conducive to the development of mosquitoes, thus to malaria transmission [17]. These two reasons have guided the choice of Douala for the study. In order to preserve the reputation of these enterprises, they were assigned; so their names are not disclosed (Table 1).

This cross-sectional study was conducted between February 2015 and June 2017. Prior to interviews, officials of each enterprise were met and given clear explanation of the aims of the study. After authorization from the management, dates were set for interviews and the communication unit was charged with disseminating the information to employees. Only employees who gave written inform consent were enrolled in the study. A pretested structured questionnaire was used to document the answers from respondent.

### 2.2. Study Population and Eligibility Criteria

The study population was made up of employees. Inclusion criteria were (i) being an employee of the enterprise and (ii) having signed an informed consent form (Additional file 1). Conversely, any participant who did not meet up with at least one of the above-mentioned criteria was excluded from the study.

### 2.3. Determination of Sample Size

The sample size was determined using Lorentz's formula as follows: *N* = *p*∗(1–*p*)∗*Z*^2^/*d*^2^, where *N* is the estimated minimum sample size, *p* is the proportion of employees having a good level of knowledge on malaria, *Z* is the statistic for desired level of confidence (1.96 for 95% confidence level), and *d* is the accepted margin of error (5%). Due to the absence of data on KAP studies among employees in Cameroon, a level of knowledge of 50% was assumed; thus, the minimum sample size was 384. In total, 2705 employees were included in the study.

### 2.4. Collection of Data of Interest

Data were collected using a structured questionnaire (Additional file 2). The first part of the questionnaire was about demographic variables which included age, gender, area of residence, level of education, enterprises, and professional category. The second part was designed to capture knowledge, attitudes, and practices towards malaria including recognition of signs and symptoms, transmission, pathogen, prevention, treatment and management, perception of malaria, and date of last malaria attack. The questionnaire was written in the two official languages (French and English) (Additional file 2). To insure its reliability and eliminate bias of understanding, the questionnaire was pretested on 15 randomly chosen individuals and updated accordingly before data collection. Interviewers were trained on all aspects of data collection. Each interview lasted 15 to 20 minutes. The participants were informed on the importance of answering questions honestly. The principal investigator supervised data collectors on a daily basis, and the filled forms were scrutinized thoroughly every day.

### 2.5. Ethical Considerations

Ethical clearance was issued by the Institutional Review Board (IRB) of the University of Douala (under No. CE218 DU/268/05/2019/T). Further authorizations were obtained from the management of enterprises. The study was carried in accordance with guidelines for human experimental models in clinical research as stated by the Cameroon Ministry of Public Health. The aim of the study was clearly explained to employees in order to get their informed consent; employees who did not consent to participate were excluded from the study. Confidentiality of personal information was insured throughout the study. After interviews, employees were educated on malaria prevention and control based on their misconceptions.

### 2.6. Statistical Analysis

All data were keyed into an Excel spreadsheet and then analyzed with the statistical package for social science (SPSS) version 16 for Windows (SPSS Inc., Chicago, IL, USA). Goodness-of-fit, chi-square independence (*χ*^2^) and Fisher's exact tests were used to compare proportions. Univariate and multivariate logistic regression models were used to identify factors associated with knowledge of malaria namely mode of transmission, causative agent, and use of paracetamol as antimalarial drug. Multimodal regression was used to identify the factors associated with the utilization of at least one preventive method. Odds ratios (OR) and 95% confidence intervals (95% CI) were also computed. Statistical significance was set at *P* < 0.05.

## 3. Results

### 3.1. Sociodemographic Characteristics of the Employees

Sociodemographic information of respondents are presented in Table 2. Most of the respondents were male (83.1%). Half of the participants were aged 36-59 years old (50.0%), and a statistically significant difference was found in proportions of different age groups (*p* = 0.012). The mean age of respondents was 37.33 ± 9.78 years old (range 19-87). Based on the level of education, respondents having completed who completed upper secondary were the most represented (28.4%) while 11.9% of respondents were holders of master degrees or equivalent and above. Employees were mainly workers (80.4%) while 2.4% were managers. Slightly above half of the respondents, 1345 (50.6%) were living in the third district of Douala. 28.7% of interviewees were living in households with 3-4 members (Table 2).

### 3.2. Knowledge on Malaria

#### 3.2.1. Signs and Symptoms

The employees' responses to questions on malaria signs and symptoms and route of transmission are summarized in Table 3. More than 90% of employees knew at least one sign or symptom of malaria. Fever was the most cited sign (*n* = 1750, 64.7%) followed by headache (39.1%), body pain (31.9%), and chills (22.8%).

#### 3.2.2. Routes of Transmission

The majority of employees interviewed (90.7%, *n* = 2452) knew that mosquitoes were responsible for malaria transmission. Others (9.3%) gave wrong answers as they attributed malaria transmission to various factors (bite of phlebotomy, consumption of dirty water, cold weather, lack of sport exercise, and consumption of sugar).

### 3.3. Causative Agent

Less than one in ten respondents (*n* = 255, 9.4%) knew that *Plasmodium falciparum* is the pathogen responsible for malaria infection. However, wrong answers (8.91%) were recorded, namely, *Entamoeba histolytica*, mosquitoes, *Anopheles* mosquitoes, flies (*Glossina palpalis*), insects, insalubrity, and consumption of dirty fruits/water.

#### 3.3.1. Drugs for the Treatment of Malaria

**Figure 1:**
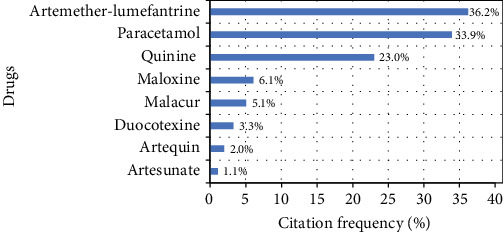
Answers of participants about drugs used for treating malaria episodes.

More than half of the participants knew the name of at least one antimalarial drug (52.0%, *n* = 2705). The most cited malaria drugs were Artemether-Lumefantrine (Coartem®) and quinine with 36.2% and 23.8% citation frequencies, respectively (Figure 1). Paracetamol was cited by 914 (33.9%) respondents.

### 3.4. Date of Last Malaria Attack

Sixteen percent of employees got their last malaria attack less than a month ago. The others reported last malaria attack 1-6 months (28.9%), 7-11 months (18.9%), or ≥12 months (25.3%) ago. It should be noted that 2.1% employees said they never had malaria.

### 3.5. Attitudes towards Malaria

Almost all (98.2%) respondents agreed with the fact that malaria is a dangerous disease, and the main reasons were that “it kills” (86.2%), “it causes anemia” (1.04%), and “it decreases productivity” (0.3%). Other reasons such as “it is the cause of absenteeism” (0.0005%) and “it weakens the body” (0.006%) were also reported.

### 3.6. Malaria Prevention Methods Used by the Respondents

Eight preventive methods were reported by the respondents. The most reported were ITNs, IRS, and environmental sanitation (Additional file 3). The majority of respondents (57%) used one preventive method only while the rest used two (30.1%), three (4.8%), and four (0.4%) methods. The combinations ITN+IRS (8.4%) and ITN+long-sleeved clothes (8.5%) were the most reported by those using two preventive methods (Additional file 3).

### 3.7. Management of Malaria Cases by Employees'

Sixty-three percent of respondents declared going to hospital when they feel having malaria (Table 4). The rest of employees declared mainly managing malaria by buying drugs from pharmacy (26.3%) and street vendors 338 (12.9%). To be noted, a small fraction of interviewees were resorting to traditional medicine (8.6%, Table 4). The reason for buying drugs from street vendors was mainly associated with price difference between drugs from pharmacy and those from street vendors.

### 3.8. Association between Sociodemographic Factors and Knowledge of Malaria

Level of education, socioprofessional category, and enterprise were significantly associated with the knowledge on the causative agent of malaria. Employees having completed lower and upper secondary education were nearly 4 times (OR = 3.51, *P* < 0.0001) and 8 times (OR = 7.59, *P* < 0.0001) more knowledgeable on the causative agent of malaria compared with those with no formal studies or with primary education only (Tables 5 and 6). In addition, the level of knowledge was 14 times higher (OR = 14.30, *P* < 0.0001) in those holding bachelor's degree and 13 times (OR = 12.81, *P* < 0.0001) higher in those holding master's degree or above. Besides, managers were more knowledgeable than laborers (OR = 1.46, *P* = 0.039) while employees from enterprises 4 and 6 were more knowledgeable on the causative agent than those from enterprise 1 (OR = 4.06, *P* = 0.004, and OR = 3.49, *P* = 0.017, respectively).

Three variables were also significantly associated with knowledge on malaria transmission (Tables 5 and 6). Employees with no formal studies/primary level of education were less knowledgeable on malaria transmission than those having completed secondary and university studies. For instance, holders of bachelor's or master' degree and above were, respectively, nearly 4 times (OR = 3.77, 95% CI 2.13-6.70, *P* < 0.0001) and 6 times (OR = 6.42, 95% CI 2.60-15.90, *P* < 0.0001) more knowledgeable on the route of malaria transmission than those having no formal studies or competed primary studies. Respondents from the Douala 3 District were 1.63 times more knowledgeable on malaria transmission than those from Douala 1 (OR = 1.63, *P* = 0.026). Regarding enterprises, employees from enterprises 7, 10, 11, and 14 were more knowledgeable than those from enterprise 1 (*P* = 0.023, *P* = 0.013, *P* = 0.022, and *P* = 0.017, respectively; Tables 5 and 6).

The area of residence was the only factor associated with use of paracetamol as a single antimalarial drug. The risk of using paracetamol only as antimalarial drug was lesser in individuals living in the Douala 4 District (OR = 0.50, *P* = 0.016) than their counterparts living in Douala 1 District (Tables 5 and 6).

The level of education and socioprofessional category were significantly associated with the use of preventive methods as presented in Tables 5 and 6. Chances for using one preventive method were higher in employees having completed lower and upper secondary studies, respectively, as compared with those having completed primary or no formal studies (OR = 1.63, *P* = 0.027, and OR = 1.76, *P* = 0.041, respectively). Managers were about 3 times likely to use a preventive method compared with laborers (OR = 2.92, *P* = 0.026).

## 4. Discussion

Most of the respondents in this study (94.1%) knew at least one symptom of malaria and 91% mentioned at least three symptoms. This high level of knowledge agrees with previous studies in Cameroon [10, 18]. Douala is a setting of perennial transmission of malaria; thus, individuals living in this areas have likely faced a malaria episode at least once in their life [10, 16].

The knowledge of malaria causative agent was low (9.4%). This result is in line with that from Kojom and Lehman [10] in Cameroon and Mazigo et al. [18] in Tanzania who found the level of knowledge of 14.3% and 6%, respectively. During health campaigns and other sensitization activities, the malaria causative agent is usually not made mention of, focus being on the vector [10]. This can explain why many employees confused causative agent with malaria vector.

More than half of respondents (52%) had good knowledge of treatment of malaria. ACTs were most reported, and this is consistent with previous studies in the country [10, 18]. Cameroon has adopted ACTs in 2004 for the treatment of uncomplicated malaria [19]. Besides, one-third (33.9%) of employees cited paracetamol for malaria treatment. This can be explained by the fact that this paracetamol is usually included in the package prescribed for the treatment [10]. Taking paracetamol as an antimalarial can result in the aggravation of malaria with complications such as kidney failure [10].

Nearly 17% of employees reported on a malaria episode less than a month ago, lower than a previous report from Burkina Faso (32%) in 1988 [20]. The difference may be due to the fact that from 1988 to 2016, great efforts have been made for malaria prevention and control, including sensitization and mass distribution of bed nets, resulting in lower prevalence of malaria. Besides, 70% of employees reported having at least one malaria attack in the past 12 months; Douala is located in a perennial transmission area with nearly 300 infective bites per man per year [21]. Thus, malaria transmission is high and the population exposed to multiple malaria episodes.

Malaria was perceived dangerous by most of the respondents (98.2%) while 1.8% was somehow indifferent. This latter category may be at risk of suffering from severe malaria as they may not bother to prevent or seek for treatment on time [22, 23]. The level of awareness regarding signs and symptoms of malaria, as well as the perceived seriousness of the disease reported in this study, was higher than previously found in the Littoral and Southwest regions of Cameroon [22, 23]. This could be probably due to the fact that people in malaria endemic areas are more likely to be more knowledgeable about disease and its consequences than those in malaria-free or low-endemic areas.

Most of the respondents knew at least one malaria prevention method. Three preventive measures were mostly used by employees namely bed nets, insecticide spray, and environmental sanitation. This result is consistent with that of previous studies in the same setting [23]. ITNs are the main preventive methods used by respondents (77.23%); this key tool for malaria control serves as physical and chemical barrier protecting users from infective mosquito bites [24]. The ITN use rate was higher than that of studies conducted in the Littoral and Centre regions [3, 25]. Conversely, this proportion is lower than that found in other Cameroonian studies and WHO-recommended target (80%) [1, 11, 26]. This difference could be due to discrepancies in study design, study period, and population. In addition, these discrepancies could also be attributable to that fact employees received bed nets at work as a result of health promotion policies implemented in enterprises where they work as reported previously in Nigeria [9]. It is plausible that the true proportion of employees using ITNs could be lower than that estimated in the present study since a self-report-based approach was used to determine the rate of ITN use. In a recent meta-analysis, Krezanoski and colleagues showed that that approach had overestimated ITNs adherence by >13% as compared to evidence-based approach [27].

However, the use of ITN insecticide spray and environmental sanitation do not guarantee a 100% protective effect especially against insecticide-resistant and/or outdoor-biting mosquitoes. Thus, it is crucial to associate complementary methods such as larval control and tracking of asymptomatic carriers of malaria parasites in order to efficiently control and/or eliminate malaria in a given area [3, 11]. Previous studies in Tanzania have shown reduction in malaria transmission following the implementation of larval control interventions [28, 29].

Other employees were using traditional medicine (herbs and parts of plants), and this could be linked to sociocultural beliefs and practices [10, 30]. To be noted, a fraction of employees said they go to pharmacy to seek for treatment. This was revealed in previous reports from the same areas, which also outlined the ease with which drugs could be obtained in pharmacies, without a medical prescription [3].

Besides, some employees were buying antimalarial treatment from street vendors. Previous studies in the Littoral and Northwest regions of Cameroon described the same phenomenon [23, 31]. The main reason given by respondents was their cheaper prices, compared with that of drugs available in hospital or pharmacy [32]. Street drugs are a major public health problem as many studies have shown the poor quality of these drugs, especially antimalarial, due to their questionable origin and poor storage conditions [33, 34]. There is also a concern about information given to their clients by vendors, regarding the type and dose of drugs [35]. Thus, the use of such drugs can increase the risk of complications in individuals resorting to street drugs and the appearance and spread of antimalarial drug resistance [10]. Artemether-Lumefantrine (AL) was the most cited antimalarial drug mainly under its commercial name Coartem®; authors have reported poor quality Artemether-Lumefantrine (AL) 20/120 mg sold illegally in Cameroon [33].

Level of education was significantly associated with knowledge of malaria transmission and causative agent. Higher educational level significantly improved the knowledge of malaria transmission and causative agent. This can be explained by the fact that the more educated you are, the better you can access to knowledge and understand information on the disease [36]. This finding is in line with previous studies in the towns of Douala and Yaoundé [10, 34]. The impact of education has also been demonstrated on the use of preventive method as the chances of using one preventive methods were higher in employees with higher levels of education.

It was also found that the chances for using three preventive methods were higher among managers compared to laborers. This could be due to income discrepancies between the two groups, managers having easier access to a variety of preventive methods.

## 5. Conclusions

This study revealed a high level of knowledge of signs and symptoms, mode of transmission, and drugs used for treating malaria among employees. They were aware of the dangers of malaria and used prevention methods, especially ITNs. However, some misconceptions and ill behavior still exist among them especially in those with a low level of education. Age, area of residence, socioprofessional category, and enterprises have significant influence on knowledge and practices of employees regarding malaria transmission, causative agent, and treatment and prevention practices. Hence, there is a need for developing sensitization strategies for employees, especially those with lower levels of education. Commitment of enterprises could foster malaria control in Cameroon.

## Figures and Tables

**Table 1:** Enterprises distributed by branch of activity and codes.

Branch of activity	Code of enterprises	Location
Cocoa and coffee export	ENT 1	Douala 1
Security	ENT 2, ENT 5, ENT 7	Douala 1
Electricity	ENT 3	Douala 1
Food processing	ENT 4	Douala 3
Employment agency	ENT 6	Douala 1
Hydraulic and drilling	ENT 8	Douala 1
Public hygiene and sanitation	ENT 9	Douala 3
Hotel	ENT 10	Douala 1
Construction and public works	ENT 11	Douala 3
Manufacturing and selling mattresses and foam	ENT 12	Douala 3
Distribution of petroleum products	ENT 13	Douala 1
Car dealership	ENT 14	Douala 3

**Table 2:** Demographic characteristics of the participants with respect to study enterprises.

Characteristics	Categories	Total (*n* = 2705)	*P* value
Gender	Female	458 (16.9)	<0.001^∗^
	Male	2247 (83.1)	

Age (years)	19-36	1307 (48.3)	
	36-60	1351 (50.0)	0.012^∗^
	≥60	47 (1.7)	

Level of education	None/primary	456 (16.9)	
	Lower secondary	656 (24.3)	
	Upper secondary	769 (28.4)	<0.001^∗^
	Bachelor's degree	505 (18.6)	
	Master's degree and above	319 (11.8)	

Professional category	Workers	2176 (80.4)	
	Agent-control	463 (17.2)	<0.001^∗^
	Managers	66 (2.4)	

Work time	Day	1522 (56.3)	<0.001^∗^
	Night	1183 (43.7)	

Residence	Douala 1	325 (12.2)	
	Douala 2	221 (8.3)	
	Douala 3	1345 (50.6)	<0.001^∗^
	Douala 4	174 (6.5)	
	Douala 5	568 (21.4)	
	Outside Douala	27 (1.0)	

Household size^#^	≤2	546 (20.6)	
	3-4	761 (28.7)	0.048^∗^
	5-6	735 (27.7)	
	≥7	612 (23.0)	

Data are presented as frequency (percentage). ENT: enterprise. ^#^Missing data. The goodness-of-fit chi-square test was used to compare proportions. ^∗^Significant at *P* < 0.05.

**Table 3:** Respondents' knowledge on signs and symptoms of malaria.

Answers	Frequency	Percentage (%)
Number of symptoms cited		
0	159	5.9
1	793	29.4
2	1147	42.5
3	516	19.1
4	77	2.9
5	9	0.3
Symptoms cited^∗^		
Fever	1750	64.7
Headache	1058	39.1
Body pain	864	31.9
Chills	617	22.8
Fatigue	539	19.9
Poor appetite	505	18.7

^∗^Frequency is more than the total number of employees (2705) as an employee who can give more than one answer.

**Table 4:** Home management of malaria cases by employee's households.

Responses	Citation frequency	Percentage (%)
Hospital	1624	63.0
Pharmacy	690	26.3
Street drugs	338	12.9
Plants (traditional medicine)	225	8.6
Hospital and pharmacy	170	6.5
Hospital and street drugs	41	1.6
Pharmacy and street drugs	41	1.6

**Table 5:** Factors associated with knowledge of the causative agent, route of malaria transmission, the use of paracetamol as antimalarial drug, and the number of preventive methods used.

Factors	Causative agent of malaria				Routes of transmission			Use of paracetamol as antimalarial		
	*N*	*n* (%)	OR (95% CI)	*P* value	*n* (%)	OR (95% CI)	*P* value	*n* (%)	OR (95% CI)	*P* value
Gender										
Female	458	58 (12.7%)	1		430 (93.9)	1		88 (19.2%)	1	
Male	2247	197 (8.8%)	0.99 (0.70-1.39)	0.938	2039 (90.7)	0.88 (0.57-1.37)	0.579	393 (17.5%)	0.85 (0.64-1.12)	0.245
Age (years)										
19-36	1307	134 (10.3%)	1		1195 (91.4)	1		230 (17.6%)	1	
36-60	1351	119 (8.8%)	0.98 (0.73-1.30)	0.865	1236 (91.5)	1.13 (0.85-1.51)	0.394	240 (17.8%)	1.08 (0.88-1.33)	0.471
≥60	47	2 (4.3%)	0.63 (0.14-2.76)	0.537	38 (80.9)	0.56 (0.24-1.30)	0.180	11 (23.4%)	1.54 (0.76-3.12)	0.235
Level of education										
None/primary	456	5 (1.1%)	1		375 (82.2)	1		91 (20.0%)	1	
Lower secondary	656	28 (4.3%)	3.51 (1.34-9.23)	0.0109^∗^	586 (89.3)	1.74 (1.22-2.50)	0.002^∗^	114 (17.4%)	0.86 (0.63-1.18)	0.361
Upper secondary	769	70 (9.1%)	7.59 (3.00-19.19)	<0.0001^∗^	712 (92.6)	2.29 (1.56-3.38)	<0.0001^∗^	127 (16.5%)	0.86 (0.62-1.18)	0.344
Bachelor's degree	505	94 (18.6%)	14.30 (5.57-36.72)	<0.0001^∗^	484 (95.8)	3.77 (2.13-6.70)	<0.0001^∗^	92 (18.2%)	1.02 (0.71-1.49)	0.897
Master's degree and above	319	58 (18.2%)	12.81 (4.76-34.49)	<0.0001^s^	312 (97.8)	6.42 (2.60-15.90)	<0.0001^∗^	57 (17.9%)	1.11 (0.70-1.76)	0.447
Professional category										
Workers	2176	158 (7.3%)	1		1956 (89.9)	1		394 (18.1%)	1	
Managers	529	97 (18.3%)	1.46 (1.02-2.10)	0.039^∗^	513 (97.0)	1.54 (0.83-2.86)	0.173	87 (16.4%)	0.88 (0.63-1.23)	0.667
Residence										
Douala 1	325	24 (7.4%)	1		285 (87.7)	1		61 (18.8%)	1	
Douala 2	221	18 (8.1%)	1.64 (0.83-3.26)	0.154	189 (85.5)	1.11 (0.65-1.89)	0.704	38 (17.2%)	0.79 (0.50-1.23)	0.447
Douala 3	1345	124 (9.2%)	1.51 (0.92-2.49)	0.102	1237 (92.0)	1.63 (1.06-2.50)	0.026^∗^	252 (18.7%)	0.96 (0.69-1.34)	0.811
Douala 4	174	20 (11.5%)	1.76 (0.91-3.40)	0.093	160 (92.0)	1.63 (0.84-3.16)	0.148	18 (10.3%)	0.50 (0.28-0.88)	0.016^∗^
Douala 5	568	64 (11.3%)	1.31 (0.78-2.20)	0.309	532 (93.7)	1.51 (0.91-2.49)	0.110	99 (17.4%)	0.91 (0.63-1.31)	0.597
Out of Douala	27	1 (3.7%)	0.50 (0.06-4.07)	0.521	25 (92.6)	1.49 (0.33-6.75)	0.606	7 (25.9%)	1.48 (0.59-3.72)	0.404
Enterprises										
ENT 1	104	5 (4.8%)	1		89 (85.6)	1		13 (12.5%)	1	
ENT 2	78	4 (5.1%)	1.77 (0.44-7.05)	0.421	64 (82.1)	1.05 (0.46-2.39)	0.916	12 (15.4%)	1.20 (0.51-2.83)	0.675
ENT 3	44	5 (11.4%)	1.92 (0.51-7.19)	0.334	43 (97.7)	5.17 (0.65-41.21)	0.121	9 (20.5%)	1.76 (0.68-4.54)	0.243
ENT 4	276	56 (20.3%)	4.06 (1.54-10.70)	0.004^∗^	255 (92.4)	1.59 (0.76-3.33)	0.221	56 (20.3%)	1.63 (0.84-3.17)	0.148
ENT 5	275	10 (3.6%)	1.20 (0.39-3.70)	0.748	228 (82.9)	1.20 (0.62-2.35)	0.587	57 (20.7%)	1.84 (0.95-3.57)	0.071
ENT 6	113	24 (21.2%)	3.49 (1.25-9.81)	0.017^∗^	108 (95.6)	2.51 (0.85-7.46)	0.097	21 (18.6%)	1.50 (0.70-3.22)	0.299
ENT 7	178	16 (9.0%)	2.33 (0.81-6.71)	0.117	166 (93.3)	2.60 (1.14-5.92)	0.023^∗^	31 (17.4%)	1.53 (0.76-3.11)	0.235
ENT 8	132	8 (6.1%)	1.18 (0.37-3.80)	0.785	116 (87.9)	1.22 (055-2.72)	0.627	17 (12.9%)	0.99 (0.46-2.17)	0.989
ENT 9	408	21 (5.1%)	1.66 (0.59-4.65)	0.338	363 (89.0)	1.69 (0.86-3.31)	0.125	90 (22.1%)	188 (0.99-3.58)	0.054
ENT 10	147	9 (6.1%)	1.00 (0.31-3.20)	0.997	141 (95.9)	3.55 (1.30-9.69)	0.013^∗^	28 (19.0%)	1.54 (0.75-3.17)	0.236
ENT 11	104	13 (12.5%)	2.57 (0.86-7.70)	0.092	100 (96.2)	3.89 (1.21-12.53)	0.022^∗^	13 (12.5%)	0.95 (0.41-2.18)	0.900
ENT 12	65	2 (3.1%)	0.69 (0.13-3.72)	0.661	61 (93.8)	2.80 (0.87-9.07)	0.085	10 (15.4%)	1.16 (0.47-2.83)	0.749
ENT 13	143	18 (12.6%)	1.77 (0.44-7.05)	0.421	135 (94.4)	1.46 (0.57-3.74)	0.431	15 (10.5%)	0.77 (0.34-1.71)	0.514
ENT 14	638	64 (10.0%)	1.86 (0.72-4.85)	0.201	600 (94.0)	2.26 (1.15-4.45)	0.017^∗^	109 (17.1%)	1.29 (0.69-2.42)	0.427

OR : odds ratio; 95% CI: confidence interval at 95%. Multivariate logistic regression model was used to compute OR. ^∗^Statistically significant at *P* < 0.05.

**Table 6:** Factors associated with the number of preventive methods used.

	One method used		Two methods used		Three methods used	
Factors	OR (95% CI)	*P* value	OR (95% CI)	*P* value	OR (95% CI)	*P* value
Gender						
Female	1		1		1	
Male	0.96 (0.64-1.44)	0.835	0.81 (0.50-1.29)	0.369	0.84 (0.34-2.07)	0.700
Age (years)						
19-36	1		1		1	
36-60	0.95 (0.71-1.28)	0.741	1.01 (0.72-1.43)	0.941	1.24 (0.60-2.54)	0.558
≥60	2.18 (0.51-9.34)	0.294	2.54 (0.52-12.48)	0.251	2.68E-7 (0.00-?)	0.996
Level of education						
None/primary	1		1		1	
Lower secondary	1.31 (0.87-1.97)	0.201	1.14 (0.69-1.86)	0.614	0.18 (0.02-1.65)	0.127
Upper secondary	1.63 (1.05-2.51)	0.027^∗^	1.56 (0.94-2.60)	0.087	2.24 (0.67-7.55)	0.191
Bachelor's degree	1.76 (1.02-3.08)	0.041^∗^	1.67 (0.88-3.14)	0.114	2.31 (0.58-9.21)	0.237
Master's degree and above	1.22 (0.64-2.34)	0.542	1.36 (0.64-2.89)	0.427	1.01 (0.20-5.06)	0.991
Professional category						
Workers	1		1		1	
Managers	1.14 (0.69-1.88)	0.597	1.37 (0.78-2.41)	0.227	2.92 (1.13-7.54)	0.026^∗^
Residence						
Douala 1	1		1		1	
Douala 2	0.68 (0.38-1.20)	0.187	1.37 (0.78-2.41)	0.277	0.18 (0.02-1.66)	0.131
Douala 3	1.11 (0.69-1.78)	0.669	0.70 (0.35-1.40)	0.319	1.33 (0.47-3.76)	0.587
Douala 4	1.03 (0.52-2.05)	0.929	1.10 (0.63-1.90)	0.742	0.24 (0.03-2.24)	0.211
Douala 5	0.97 (0.58-1.64)	0.897	1.33 (0.61-2.89)	0.473	0.53 (0.16-1.75)	0.301
Out of Douala	0.63 (0.17-2.34)	0.494	0.70 (0.38-1.27)	0.238	8.78E-8 (0.00-?)	0.995
Enterprises						
ENT 1	1		1		1	
ENT 2	0.93 (0.36-2.43)	0.885	0.53 (0.16-1.75)	0.297	1.31E-7 (0.00-?)	0.995
ENT 3	4.23 (0.51-34.75)	0.179	3.66 (0.39-33.98)	0.254	3.39 (0.14-82.97)	0.454
ENT 4	1.18 (0.52-2.66)	0.688	0.90 (0.35-2.32)	0.825	1.49 (0.25-8.77)	0.658
ENT 5	0.93 (0.43-2.02)	0.853	0.78 (0.31-1.94)	0.589	0.58 (0.07-4.91)	0.618
ENT 6	1.10 (0.40-3.01)	0.855	1.63 (0.53-4.98)	0.391	1.91 (0.26-14.21)	0.525
ENT 7	1.42 (0.58-3.51)	0.443	1.77 (0.64-4.91)	0.269	3.90 (0.63-24.17)	0.143
ENT 8	1.14 (0.46-2.82)	0.778	0.78 (0.27-2.28)	0.655	0.78 (0.09-6.81)	0.821
ENT 9	1.37 (0.63-2.99)	0.429	1.37 (0.56-3.37)	0.493	0.39 (0.04-3.32)	0.385
ENT 10	1.27 (0.51-3.17)	0.607	1.07 (0.37-3.08)	0.897	0.40 (0.03-5.19)	0.48
ENT 11	2.45 (0.79-7.61)	0.121	0.56 (0.13-2.35)	0.429	8.06E-8 (0.00-?)	0.995
ENT 12	0.98 (0.33-2.90)	0.963	1.64 (0.49-5.52)	0.421	1.45E-7 (0.00-?)	0.994
ENT 13	0.97 (0.40-2.36)	0.955	0.48 (0.16-1.41)	0.181	0.63 (0.08-4.72)	0.651
ENT 14	1.67 (0.78-3.54)	0.183	0.88 (0.37-2.13)	0.784	0.86 (0.15-4.94)	0.869

OR: odds ratio; 95% CI: confidence interval at 95%. Multimodal logistic regression model was used to compute OR. ?: no value available. ^∗^Statistically significant at *P* < 0.05.

## Data Availability

All data generated or analyzed during this study are included in this published article.

## Abbreviations

ACT: Artemisinin-based combination therapy
AL: Artemether-Lumefantrine
CCA/SIDA: *Coalition de la Communauté des Affaires contre le SIDA, la tuberculose et le paludisme*
CI: Confidence interval
IRB: Institutional Review Board
IRS: Indoor residual spraying
ITN: Insecticide-treated net
KAP: Knowledge, attitudes, and practices
LLINs: Long-lasting insecticide-treated nets
NGO: Nongovernment organization
OR: Odds ratio
RBM: Roll Back Malaria
SPSS: Statistical package for social sciences
URED: University-Research-Development-Environment
WHO: World Health Organization.

## Supplementary Materials

Supplementary Materials Additional File 1: informed consent form (English and French versions). Additional File 2: questionnaire form used to document data of interest (English and French versions). Additional File 3: malaria preventive methods used by employees.
