# Supplementary material for: Knowledge, Attitude, and Practices towards Malaria among Employees from Enterprises in the Town of Douala, Cameroon
Source: Biomed Res Int. 2020 Jun 26;2020:8652084. doi: 10.1155/2020/8652084 (PMC7335393; doi:10.1155/2020/8652084)
Supplement: Supplementary Materials — Additional File 1: informed consent form (English and French versions). Additional File 2: questionnaire form used to document data of interest (English and French versions). Additional File 3: malaria preventive methods used by employees. [file 8652084.f1.docx]

**INFORMED CONSENT (ENGLISH VERSION)**

Theme of the study: **Knowledge, Attitudes and Practices on malaria prevention and control among employees in Cameroon** certify that I have read (or that someone has read for me) the notice explaining the purpose of the study project and the expectations towards me. The opportunity was offered to me to ask all my questions and satisfactory answers were provided to me. I received a copy of this document. I understand what is expected of me if I agree to participate in the study. I also know that I can suspend my participation in this study at any time without any risk or prejudice. It has also been explained to me that all the personal data that I will provide will be kept in the confidentiality and the respect of the medical secret. **BY SIGNING THIS DOCUMENT, I AGREE TO FULL GRE PARTICIPATE IN THE STUDY IT PRESENTED**.

**Name of Participant** ________________________________ ______________________

Signature of the Participant Date

**INVESTIGATOR**

I explained the study and answered all the questions of the participant. I believe that she understood the information in the notice and that she freely agrees to participate in this study.

________________________________________

**Name of the Investigator**

________________________________________ __________________________

Signature of the Investigator Date (the same as that of the participant)

**CONSENTEMENT ECLAIRE (FRENCH VERSION)**

Thème de l'étude: **Connaissances, attitudes et pratiques des employés en rapport avec le paludisme au Cameroun** Certifie que j'ai lu (ou que quelqu'un a lu pour moi) la description du sujet de l'étude et les attentes à mon égard. L'occasion m'a été offerte de poser mes questions et réponses. J'ai reçu une copie de ce document. Je comprends ce que l'on attend de moi si j'accepte de participer à l'étude. Je sais également que je peux suspendre ma participation à cette étude à tout moment sans risque ni préjudice. On m'a expliqué qu'il sera gardé dans la confidentialité et le respect du secret médical. En signant ce document, j'accepte de participer pleinement à l'étude présentée.

**Nom du participant** ___________________________________ __________________

Signature du participant Date

**INVESTIGATEUR**

J'ai expliqué l'étude et répondu à toutes les questions du participant. Je crois qu'elle a compris l'information contenue dans cette brochure et a accepté de participer à cette étude.

________________________________________

**Nom de L’INVESTIGATEUR**

________________________________________ ______________________________

Signature de L’INVESTIGATEUR Date

**QUESTIONNAIRE (ENGLISH VERSION)**

**Enterprise ………**

**N ...... Neighborhood ................ Age ................. Sex ......................**

**Level of study ...................................... Occupation ............................ Tel .............................**

1. What are the signs of malaria you know?.....................................................................................................

2. What is the date of your last malaria attack .................................................................................................

3. What is the mode of transmission of malaria ...............................................................................................

4. What method of malaria prevention do you practice? .................................................................................

5. What other methods of prevention do you know .........................................................................................

6. Is malaria dangerous? Yes or No If yes, why? ............................................................................................

7. Do you often work at night? Yes ............... No…….

8. How do you protect yourself from mosquito bites? ....................................................................................

9. What is the parasite that causes malaria? ....................................................................................................

10. Number of regular use LLIN nets………………………………………………………………………..

If not why? 1) Heat 2) Forgetting 3) Allergy 4) Claustrophobia 5) Other ………………………………….

11. How many nights have you spent under a mosquito net in the last 3 days? .............................................

12. Malaria Case Management 1) Hospital 2) Pharmacy 3) Street Medic 4) Traditional Drugs? ..................

13. Medicines for malaria 1) ........................... 2) ............................. 3) ....................... 4)……………….

14. Number of people in the household ..........................................................................................................

15 Number of children in the household .........................................................................................................

**Additional file 3**

**Table** **Malaria preventive methods used by respondents**

| **Number of methods used** | **Nature** | **n** | **%** |
| --- | --- | --- | --- |
| None | / | 212 | 7.8 |
| One | 1 | 1229 | 45.4 |
|  | 2 | 101 | 3.7 |
|  | 3 | 81 | 3.0 |
|  | 4 | 36 | 1.3 |
|  | 5 | 41 | 1.5 |
|  | 6 | 49 | 1.8 |
|  | 7 | 0 | 0.0 |
|  | 8 | 4 | 0.1 |
|  | **Total** | **1541** | **57.0** |
| Two | 1 + 2 | 228 | 8.4 |
|  | 1 + 3 | 159 | 5.9 |
|  | 1 + 4 | 43 | 1.6 |
|  | 1 + 5 | 41 | 1.5 |
|  | 1 + 6 | 229 | 8.5 |
|  | 1 + 7 | 12 | 0.4 |
|  | 1 + 8 | 14 | 0.5 |
|  | 2 + 3 | 17 | 0.6 |
|  | 2 + 4 | 3 | 0.1 |
|  | 2 + 5 | 9 | 0.3 |
|  | 2 + 6 | 16 | 0.6 |
|  | 2 + 7 | 2 | 0.1 |
|  | 2 + 8 | 4 | 0.1 |
|  | 3 + 4 | 1 | 0.0 |
|  | 3 + 5 | 1 | 0.0 |
|  | 3 + 6 | 16 | 0.6 |
|  | 4 + 6 | 6 | 0.2 |
|  | 5 + 6 | 10 | 0.4 |
|  | 6 + 8 | 2 | 0.1 |
|  | **Total** | **813** | **30.1** |
| Three | 1 + 2 + 3 | 28 | 1.0 |
|  | 1 + 2 + 6 | 29 | 1.1 |
|  | 1 + 2 + 4 | 5 | 0.2 |
|  | 1 + 2 + 5 | 9 | 0.3 |
|  | 1 + 2 + 7 | 2 | 0.1 |
|  | 1 + 2 + 8 | 5 | 0.2 |
|  | 1 + 3 + 5 | 2 | 0.1 |
|  | 1 + 3 + 6 | 28 | 1.0 |
|  | 1 + 3 + 7 | 2 | 0.1 |
|  | 1 + 4 + 5 | 1 | 0.0 |
|  | 1 + 4 + 6 | 3 | 0.1 |
|  | 1 + 5 + 6 | 7 | 0.3 |
|  | 1 + 6 + 7 | 2 | 0.1 |
|  | 1 + 6 + 8 | 1 | 0.0 |
|  | 2 + 3 + 6 | 2 | 0.1 |
|  | 2 + 5 + 6 | 2 | 0.1 |
|  | 2 + 6 + 8 | 1 | 0.0 |
|  | **Total** | **129** | **4.8** |
| Four | 1 + 2 + 3 + 8 | 2 | 0.1 |
|  | 1 + 2 + 3 + 6 | 4 | 0.1 |
|  | 1 + 3 + 4 + 6 | 2 | 0.1 |
|  | 1 + 3 + 6 + 8 | 2 | 0.1 |
|  | **Total** | **10** | **0.4** |
| **TOTAL** |  | **2705** | 100.0 |

1 =Bed nets

2= Indoor residual spraying

3= Environmental sanitation

4= Preventive intermittent treatment

5=Ventilation/Air conditioner

6= Long-sleeved clothes

7= Insecticides creams/Repellents

8= Closing doors and windows

**QUESTIONNAIRE (FRENCH VERSION)**

Entreprise …………..

N……. Quartier ……………………………… Age ………………. Sexe ……………….

Niveau d’étude……………………… Profession………………………… Tel………………….

1. Quels sont les signes du paludisme que vous connaissez ? …………………………………………………

2. Quelle est la date de votre dernier accès palustre ………………………………………………………........

3. Quel est le mode de transmission du paludisme……………………………………………………………...

4. Quelle méthode de prévention du paludisme pratiquez-vous? …………….................................................. 5. Quelles autres méthodes de prévention connaissez-vous……………………………………………………..

6. Le paludisme est-il dangereux ? Oui… Non… Si Oui pourquoi ? …………………………………….

7. Travailler-vous souvent la nuit ? Oui ............... Non………

8. Comment vous protégez-vous contre les piqûres de moustiques ? ………………………………………….

9. Quel est le parasite qui cause le paludisme ? ………………………………………………………………..

10. Nombre de moustiquaire utilisée régulièrement ……………………………………………………………

Si non, pourquoi? 1) Chaleur 2) Oubli 3) Allergie 4) Claustrophobie 5) Autre ……………………….

11. Combien de nuit avez-vous passées sous une moustiquaire ces 3 derniers jours ? ……………………

12. Gestion des cas de paludisme

1) hôpital 2) pharmacie 3) Médicament de la rue 4) Médicaments traditionnels ? ………………….

13. Médicaments du paludisme 1)……………..2) …………….. 3) …………….. 4)……………………..

14. Nombre de personnes du ménage………………………………………………………………….

15 Nombre d’enfants dans le ménage ………………………………………………………………..
